# A living light bulb, ultrasensitive biodetection made easy

**DOI:** 10.1186/2045-3701-4-34

**Published:** 2014-06-30

**Authors:** Jing Shang, Xiaohu Gao

**Affiliations:** 1 Department of Bioengineering, University of Washington, Seattle, WA 98195, USA

## Abstract

A team of scientists led by Professor DW Pang at Wuhan University have developed a new class of fluorescence probes based on bacterial cells. These microbial factories manufacture semiconductor nanocrystals inside and display protein A molecules on cell surface, transforming *Staphylococcus aureus* (*S. aureus*) cells into highly fluorescent cellular beacons that can be easily adapted for detection of diverse biological targets.

## Commentary

Semiconductor nanocrystals, commonly known as quantum dots (QDs), represent a new class of nanoprobes greatly advancing and expanding the capabilities of fluorescence imaging because of their superior electrical, optical, and structural properties compared to conventional fluorophores, such as size-tunable emission color, narrow and symmetrical emission peak, large absorption coefficient throughout a wide spectrum, outstanding brightness and photostability, and extremely large Stokes shift [1,2]. Furthermore, multicolor QDs can also serve as dopants in microspheres and nanospheres for optical barcoding and imaging signal amplification [3,4].

Highly fluorescent and monodisperse QDs are often chemically synthesized via high-temperature organometallic procedure [5]. Recently, Dr. Pang’s group at Wuhan University, China, has developed a very interesting approach by taming yeast cells into a living QD synthesizer [6]. Coupling yeasts’ natural intracellular metabolic reaction of Na_2_SeO_3_ and detoxification of Cd ions, highly fluorescent CdSe QDs can be made with precisely controlled sizes and emission wavelengths. Regardless of the preparation procedure, however, the as synthesized QDs are subject to complex multistep processing, such as isolation, purification, functionalization with surface ligands, and conjugation with biomolecules (*e.g.*, antibodies), before downstream bio-imaging and -detection applications can be realized.

To address this problem, in a recent publication in ACS Nano [7], Pang’s group further advanced the microbial QD synthesizer using *S. aureus* cells, which can simultaneously produce highly fluorescent QDs inside and display Protein A on the cell surface. As a result, the whole bacterial cell is transformed into an ultrabright cellular beacon, with broad applications in ultrasensitive detection. Using pathogen detection as an example, they show detection sensitivity as low as 8.94 ng/mL (based on protein content).

The easy and sensitive detection is enabled by two key innovations. First, the application of whole cells as a fluorescent reporter eliminates the procedures for QD isolation and functionalization, which significantly simplifies assay preparation, Furthermore, the sandwich assay used in this paper maximizes the detection sensitivity by integrating the target enrichment capability of magnetic beads and signal amplification of cellular beacons [8].

Second, the protein A on *S. aureus* surface makes antibody conjugation easy. Through a simple mixing and incubation step, a variety of antibodies can be immobilized on the probe surface, enabling a broad application of the cellular beacon. It has been shown recently that although noncovalent, protein A - antibody binding is stable for at least a few hours [9,10], which is sufficient for most bio-detection assays.

In summary, the technology reported in this paper transforms cells into living light bulbs that can specifically highlight biological targets. The innovation and simplicity of this technology will stimulate further research on the use of live organisms for a wide spectrum of biomedical applications.

## Competing interests

The authors declare that they have no competing interests.

## Author’s contributions

JS and XHG wrote the article. All authors read and approved the final manuscript.
